# *purpleoid ^1^* , a classic *Drosophila* eye color mutation, is an allele of the t-SNARE-encoding gene *SNAP29*

**DOI:** 10.17912/micropub.biology.001563

**Published:** 2025-04-07

**Authors:** Derek M. Dean, Lillian E. Codd, Ruben Constanza, Xavier M. Segel

**Affiliations:** 1 Biology, Williams College, Williamstown, Massachusetts, United States

## Abstract

The *Drosophila* mutant eye color trait *purpleoid* ( *pd* ) was first observed by Calvin Bridges over a century ago. Although *pd* mutant strains have been maintained ever since, the *pd* locus has not been identified. Using complementation tests, genetic rescue, and DNA sequencing, we show that *pd ^1^* is a missense mutation in *SNAP29* ; this gene encodes a key component of the SNARE complex, which facilitates vesicle docking and fusion at cellular membranes. After describing how *pd ^1^* was mapped, we discuss ways that the mutation could be used in future studies of eye pigmentation, SNARE complex assembly, and vesicle trafficking.

**Figure 1. Mapping the Drosophila eye color trait purpleoid ( pd ) to the SNAP29 gene:**
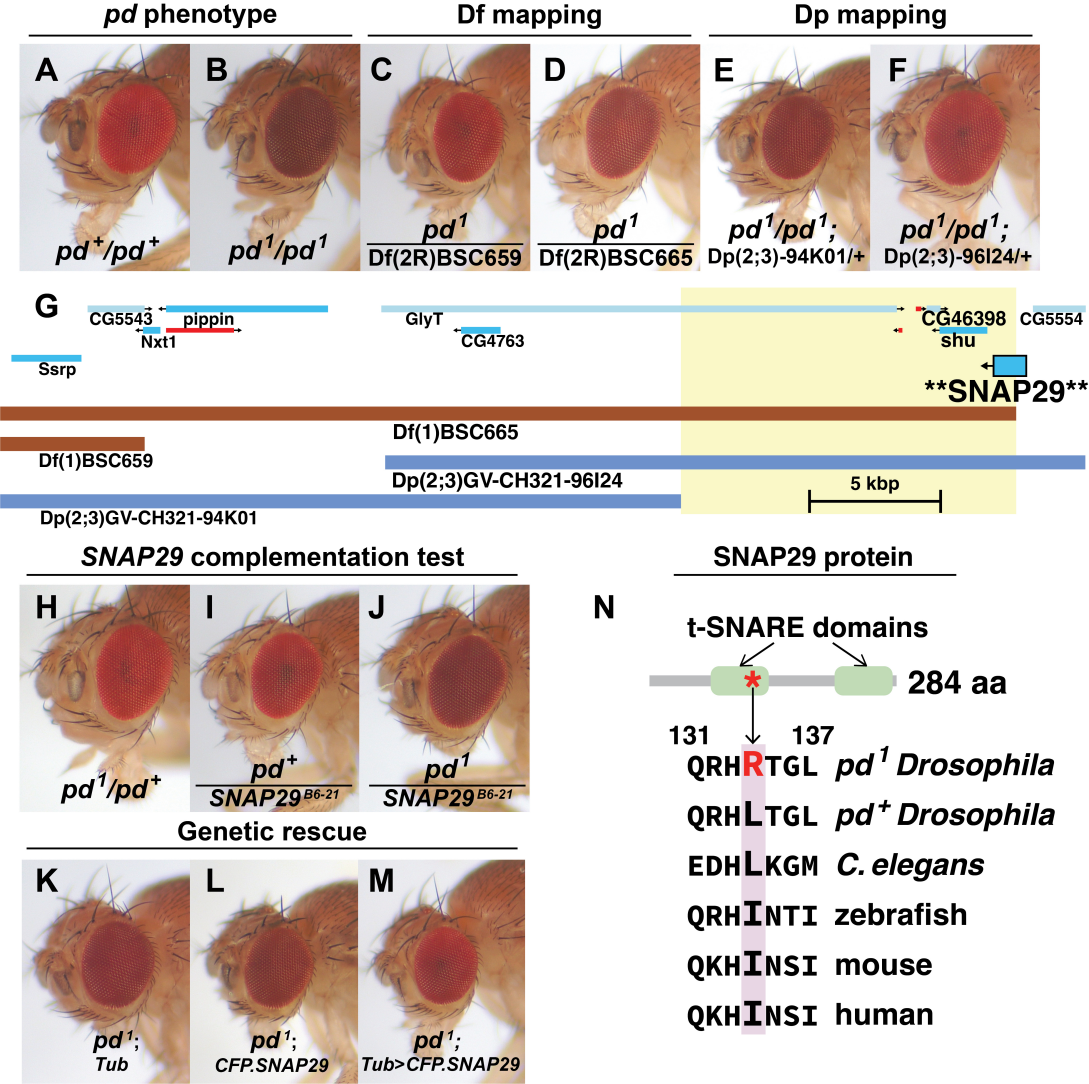
**(A)** Bright red eye of a wild-type adult *Drosophila* from the Oregon-R-P2 strain ( *pd ^+^* / *pd ^+^* ). A pseudopupil is visible in the center of the eye. **(B)** Maroon/burgundy eye of a *purpleoid ^1^* mutant ( *pd ^1^* / *pd ^1^* ). Pseudopupil is not visible. **(C)** Df(1)BSC659 complements *pd ^1^* . **(D)** Df(1)BSC665 fails to complement *pd ^1^* , suggesting that it deleted at least part of the *pd* gene. **(E)** Dp(2;3)GV-CH321-94K01 does not complement *pd ^1^* . **(F)** Dp(2;3)GV-CH321-96I24 complements *pd ^1^* , suggesting that it includes a functioning copy of the *pd* gene. **(G)** Segment of the right arm of Chromosome 2 (approximately 2R:23,805,000..23,847,000), after a JBrowse rendering. At the top of the panel, blue rectangles indicate protein-coding genes, and smaller red rectangles indicate lncRNA genes; an arrow on each gene shows 5’-3’ transcription direction. Below gene map, deficiency (Df) and duplication (Dp) ranges are shown. Based on deficiency and duplication mapping in **(C-F)** , the hypothesized *purpleoid* gene region is highlighted in yellow. Bottom right, a black scale bar shows span of 5 kilobase pairs (kbps). **(H)** *pd ^1^* / *pd ^+^* heterozygous control. **(I)** *pd ^+^* / *SNAP29 ^B6-21^* heterozygous control. **(J)** *pd ^1^* / *SNAP29 ^B6-21^* fly, showing that *SNAP29 ^B6-21^* failed to complement *pd ^1^* . **(K)** *pd ^1^* mutant expressing *Tub* -GAL4 but with no UAS transgene present (full genotype *pd ^1^* / *pd ^1^* ; *Tub* -GAL4/+). **(L)** *pd ^1^* mutant with UAS- *CFP.SNAP29* but no GAL4 construct to drive its expression (full genotype *pd ^1^* / *pd ^1^* ; +/UAS- *CFP.SNAP29* ). **(M)** *pd ^1^* mutant expressing *Tub* -GAL4 > UAS- *CFP.SNAP29* (full genotype *pd ^1^* / *pd ^1^* ; *Tub* -GAL4/UAS- *CFP.SNAP29* ); ubiquitous misexpression of CFP.SNAP29 fusion protein rescued *pd ^1^* eye color. **(N)** The *Drosophila* SNAP29 polypeptide has a predicted length of 284 amino acids and contains two target-SNARE (t-SNARE) coiled-coil protein domains (light green regions) that are widely conserved between SNAP proteins. *pd ^1^* mutants have a missense mutation in *SNAP29* that is predicted to replace a hydrophobic leucine near the end of the first t-SNARE domain with a positively-charged arginine (L134R). At the aligned position, *C. elegans* SNAP29 shares a leucine with wild type *Drosophila* SNAP29, while zebrafish, mouse, and human SNAP29 homologs all have an isoleucine, which is a conservative substitution for leucine. This suggests that the site is under selection for a hydrophobic residue. GenBank accession numbers used in BLAST alignments are listed in the Methods section.

## Description

In our Genetics class at Williams College, students have been researching the fruit fly *Drosophila melanogaster* , mapping classic mutant traits to identify their associated genes. Preliminary data are collected during classroom exercises, then students are recruited into our research lab to help verify findings, complete follow-up experiments, and coauthor our discoveries (Dean et al., 2015; Dean et al., 2020; Dean et al., 2022). In a recent iteration of the lesson plan, we worked to identify *purpleoid* ( *pd* ), an unannotated eye color gene on the right arm of chromosome 2 (2R) (Bridges, 1937; Lindsley and Zimm, 1992). Wild-type ( *pd ^+^* ) *Drosophila* have bright red eyes, while the eyes of *pd ^1^* flies have a relatively dull, maroon/burgundy color tone ( Figure 1A, B).

To map *pd ^1^* to a short chromosomal interval, *pd ^1^* flies were crossed to a series of stocks that carry molecularly defined deficiencies and duplications (abbreviated “Df” and “Dp” respectively; Cook et al., 2012; GenetiVision Corporation). Consistent with a previous report, *pd ^1^* was complemented by Df(2R)BSC659 but not by Df(2R)BSC665 ( Figure 1C, D; Kahsai and Cook, 2018). This suggested that the *purpleoid* locus is at least partially located within 2R:23811401..23844351. Seven protein-coding genes overlap this chromosomal segment: *Nxt1* , *pippin* , *GlyT* , *CG4763* , *CG46398* , *shu* , and *SNAP29* ( Figure 1G ). Subsequently, we found that *pd ^1^* eye color was not rescued by Dp(2;3)GV-CH321-94K01, but was rescued by Dp(2;3)GV-CH321-96I24 ( Figure 1E, F). Cross referencing these observations with JBrowse, it was seen that *Nxt1* and *pippin* are not covered by the complementing duplication, so they were eliminated as *pd* candidates. *GlyT* is also unlikely to be the *pd* gene because Dp(2;3)GV-CH321-96I24, the duplication that complemented *pd ^1^* , begins 166 bp downstream of the only known *GlyT* transcription start site, so it would not be expected to express *GlyT* mRNA. *CG4763* was also discounted because it is fully included on both duplications that were tested, yet only one of these duplications rescued *pd ^1^* . Thus, the duplication cross results narrowed the list of *purpleoid* candidates to *CG46398* , *shu* , and *SNAP29* (highlighted subregion in Figure 1G ). No *CG46398* mutant stocks were available for phenotypic analysis, but two different RNA-seq projects had failed to detect significant *CG46398* expression outside of the male reproductive organs, which argues against a role for *CG46398* in the eye color development of both sexes (Brown et al., 2014; Leader et al., 2018). This left *shu* and *SNAP29* as the final *purpleoid* candidates.

Fly strains carrying loss of function, homozygous-lethal mutations in *shu* and *SNAP29* were available from the Bloomington Stock Center, enabling us to conduct a round of gene-specific complementation tests. Kahsai and Cook (2018) reported that *shu ^2^* had complemented *pd ^1^* , and we replicated this result. In addition, we found that P{EPgy2} *shu ^EY08563^* , a *P* -element insertion in the 5’-UTR of *shu* , also complemented *pd ^1^* . This was further evidence that the *pd ^1^* phenotype is not due to a mutation in *shu* . Kahsai and Cook (2018) did not test *SNAP29* as the *pd* gene because they had a broader goal of mapping many different Chromosome 2 mutations, but in our experiments, we found that *SNAP29 ^B6-21^* , an amorphic *SNAP29* allele, failed to complement *pd ^1^* ( Figure 1H- J). Furthermore, ubiquitous misexpression of a UAS- *CFP.SNAP29* construct in *pd ^1^* flies rescued the eye color phenotype ( Figure 1K- M; *SNAP29 ^B6-21^* and UAS construct described in Morelli et al., 2014). Lastly, the *SNAP29* gene of *pd ^1^* flies was sequenced, and a C T G to C G G missense mutation was detected relative to *pd ^+^* controls; this would substitute a positively-charged arginine for a hydrophobic leucine in the coded protein (L134R, Figure 1N ; GenBank accession number PQ857573).

These results strongly indicate that *SNAP29* is the *purpleoid* gene, or more precisely, that the *pd ^1^* eye color phenotype is due to a mutation in *SNAP29* . Calvin Bridges first observed the *pd ^1^* phenotype in 1916 (Bridges, 1937). Since then, *pd ^1^* strains have been cultivated by researchers and stock centers, but the affected gene had not been determined until this study. Bridges’ investigations have been an integral part of undergraduate genetics curricula for many decades, so identifying the *purpleoid* gene has educational as well as historical value.

*SNAP29* ( *Synaptosomal-associated protein 29kDa* ) encodes a member of the SNARE (Soluble N-ethylmaleimide attachment protein receptor) family, a group of proteins that facilitate vesicle fusion with cellular membranes. SNAREs fall into two subcategories according to their polypeptide sequences and intracellular localizations: t-SNARE proteins such as SNAP29 are cytoplasmic and tethered to a cell membrane “ t arget”, while v-SNAREs are associated with the membranes of v esicles such as lysosomes. Binding between vesicle v-SNARE and cell membrane t-SNARE coiled-coil domains enables vesicle attachment to the target membrane. This “docking” step is followed by membrane fusion and release of vesicle content through the cellular membrane on which the vesicle had attached (Fasshauer et al., 1998; Harbury, 1998; Khvotchev and Soloviev, 2022; Yang et al., 2023).

Previous research has shown that *Drosophila* SNAREs affect vesicle trafficking during synaptic transmission and autophagy ( *e.g.* , Rao et al., 2001; Vilinsky et al., 2002; Haberman et al., 2012; Lőrincz et al., 2016; Chang et al., 2024). Our data indicate that SNARE activity affects eye pigmentation as well. This makes sense considering circumstantial evidence from past studies of lysosomal trafficking and eye color development. *Drosophila* eye color pigments (brown ommochromes and red drosopterins) are deposited in specialized lysosomes called pigment granules, and there are multiple loci that regulate lysosome biogenesis and also affect eye color. Of particular relevance, the eye color genes *deep orange* , *carnation* , and *light* encode members of the HOPS/CORVET complex, an assembly of proteins that help recruit SNAREs to the membrane. *pd ^1^* is semilethal in combination with a mutation in *deep orange* , suggesting a functional interaction between SNAP29 and the HOPS/CORVET complex (Lucchesi, 1968; Ooi et al., 1997; Simpson et al., 1997; Lloyd et al., 1998; Sevrioukov et al., 1999; Akbar et al., 2009; Solinger and Spang, 2013; Grant et al., 2016; Lőrincz et al., 2016).

*SNAP29 ^B6-21^* , the only other confirmed and available *Drosophila* *SNAP29* mutant allele, is a nonsense, amorphic mutation, making it an invaluable resource to assess the consequences of strong loss of *SNAP29* function (Morelli et al., 2014). However, *SNAP29 ^B6-21^* has limitations as an investigative tool: (1) it is homozygous lethal shortly after larval hatching, well before the adult eye has formed (however, *SNAP29 ^B6-21^* mitotic clones have been successfully generated and analyzed in the developing adult eye—see Morelli et al., 2014; Morelli et al., 2016); (2) *SNAP29 ^B6-21^* /+ heterozygotes are viable but have an eye color tone that overlaps with wild type ( Figure 1I ); and (3) the *SNAP29 ^B6-21^* mutation places a stop codon before the second t-SNARE domain, removing 116 amino acids from the C-terminus of a 284 amino acid protein (Morelli et al. *,* 2014). Such a severe truncation is likely to disrupt the folding and function of the remaining polypeptide, so *SNAP29 ^B6-21^* is unlikely to reveal the role of any specific SNAP29 domain.

On the other hand, *pd ^1^* is a missense mutation in a functionally important domain of *SNAP29* , so it offers advantages that complement those of *SNAP29 ^B6-21^* : (1) *pd ^1^* homozygotes are viable and have good fitness; (2) with proper lighting (see Methods), the eye color of *pd ^1^* homozygotes is easily distinguished from that of wild-type flies ( Figure 1A, B); and (3) *pd ^1^* is expected to have a relatively specific effect on SNAP29 structure/function because, while *SNAP29 ^B6-21^* produces a truncated protein, *pd ^1^* substitutes arginine, a charged residue, for a leucine along the hydrophobic face of a predicted α-helix within the first t-SNARE coiled coil domain ( Figure 1N ). Amino acid substitutions in the coiled coil domains of other SNARE proteins affect formation, stability, and function of the t-SNARE/v-SNARE complex (Brennwald et al., 1994; Nonet et al., 1997; Saifee et al., 1998; Rao et al. 2001). Taken together, *pd ^1^* has potential for future study. For example, it genetically interacts with mutations in *deep orange* and *garnet* , two genes that affect lysosomal delivery to membrane-bound pigment granules (Lucchesi, 1968; Lloyd et al., 1998), so the *pd ^1^* phenotype could be a useful reference point in genetic modifier screens. *pd ^1^* might also assist protein-level studies. Techniques such as coimmunoprecipitation, SDS-PAGE, Western blotting, and mass spectroscopy have successfully identified SNARE binding partners, as well as factors that affect their assembly ( *e.g.* , Itakura et al., 2012; Jiang et al., 2014; Yang et al., 2015; Li et al., 2022). This in mind, it seems feasible to compare the SNARE protein complex components of wild-type and *pd ^1^* flies. Additional fly strains could be created, each with a different missense mutation at a different site along the *SNAP29* gene, and subjected to similar analysis.

## Methods

**Fly stocks and experimental crosses:** The following fly stocks were obtained from the Bloomington *Drosophila* Stock Center (BDSC):

· Oregon-R-P2 (“ *pd ^+^* ”; BDSC 2376)

· *pd ^1^ Phm ^ll-1^* (BDSC 362)

· *w ^1118^* ; Df(2R)BSC659/SM6a (BDSC 26511)

· *w ^1118^* ; Df(2R)BSC665/SM6a (BDSC 26517)

· *w ^1118^* ; Dp(2;3)GV-CH321-94K01 (BDSC 90653)

· *w ^1118^* ; Dp(2;3)GV-CH321-96I24 (BDSC 90661)

· *cn ^1^ bw ^1^ shu ^2^ sp ^1^* /CyO, *l(2)DTS513 ^1^* (BDSC 5109; Munn and Steward, 2000)

· *y ^1^* *w ^67c23^* ; P{ *y ^+mDint2^* *w ^+mC^* =EPgy2} *shu ^EY08563^* (BDSC 17473; Bellen et al., 2004)

· w* ; P{ *ry ^+t7.2^* =neoFRT}42D *Snap29 ^B6-21^* /CyO, P{ *w ^+mC^* =GAL4-twi.G}2.2, P{ *w ^+mC^* =UAS-2xEGFP}AH2.2 (BDSC 56818)

· *y ^1^* w* ; P{ *w ^+mC^* = *tub* P-GAL4}LL7/TM3, *Sb ^1^* *Ser ^1^* (BDSC 5138; Lee and Luo, 1999)

· w* ; M{ *w ^+mC^* =UAS- *CFP.Snap29* }ZH-86Fb (BDSC 56817)

Flies were fed the modified yeast/dextrose/cornmeal diet described in Dean et al. (2020). Experimental crosses were conducted at 25°C. Mating schemes are available upon request. Experimental cross progeny were scored by at least two authors independently. Cross results were analyzed with the bioinformatic data available on FlyBase and JBrowse (Jenkins et al., 2022; Öztürk-Çolak et al., 2024).

**Photography:** A Zeiss Stemi 305 dissecting scope and its camera were used to image fly eyes. We found that *pd ^1^* eye color was much easier to discern with side lighting than with overhead lighting, so rather than using the installed overhead microscope light, two desk lamps, each fitted with a TCP Dimmable A-19 LED bulb (15W 1675 lumen 5000K), were positioned on opposite sides of the microscope stage and pointed directly towards the specimen. Adult flies 1-4 days old were collected and stored at -20°C for no more than a week, then photographed at 30X magnification under a locked camera setting (23 msec exposure, gain 0, gamma 32, contrast 8, saturation 12, RGB 21/12/26, brightness 9, sharpness 3, denoise 5). Each fly was imaged at multiple focal planes to view the entire eye. The photo series for each fly was imported into Adobe Photoshop 2021 and focal stacked (Dean et al., 2022). Photos had been intentionally underexposed with only moderate contrast to avoid loss of color information by clipping. To better represent the color balance and contrast that had been seen through the microscope, while still avoiding clipping, each stacked image was given the following post-production modifications: exposure was increased +0.7, contrast was increased +20, one sharpen filter was added, and white balance was slightly adjusted using an image of an 18% gray card (Delta Photography Supplies) that had been photographed under the same conditions as the fly specimens had been.

**DNA sequencing and sequence analysis:** Standard “squish preps” were used to extract DNA from *pd ^+^* and *pd ^1^* adult flies, two independent extractions per genotype (Gloor and Engels, 1992; Gloor et al., 1993). The *SNAP29* gene was amplified from each extract using Q5 DNA polymerase from New England Biolabs, the manufacturer’s recommended protocol, and the following PCR primers (synthesized by Integrated DNA Technologies):

· SNAP29for7: CGCTATTGCAATCGATAACTCC (forward primer, anneals to 5’-UTR)

· SNAP29rev7: AGGAATGCATTCTTAATGGCC (reverse primer, anneals to 3’-UTR)

PCR products were run through a 0.8% low melt agarose gel and extracted from gel slabs using the Monarch DNA Gel Extraction Kit (New England Biolabs). The forward and reverse strands of each product were sequenced with Sanger sequencing by the Cornell Institute of Biotechnology (Ithaca, NY), using the PCR primers listed above. Within the windows of high-quality sequence, no mismatches were seen between the forward and reverse strand readings of a sample or between the replicates of a given genotype.

The nucleotide sequence of the *pd ^1^* strain *SNAP29* gene is deposited in NCBI under GenBank accession number PQ857573. To generate the SNAP29 protein alignments shown in Figure 1N, the translation of the *pd ^1^ SNAP29* sequence was queried in a BLASTx (Camacho et al., 2009) against *Drosophila* SNAP29 protein (accession number NP_523831.1), and then the *Drosophila* SNAP29 protein sequence was queried in protein BLASTs against NP_505641.2 ( *C. elegans* ), NP_001243185.1 (zebrafish), NP_075837.3 (mouse), and NP_004773.1 (human).

No AI was used in this project for data collection, writing, editing, or figure generation.

## References

[R1] Akbar MA, Ray S, Krämer H (2009). The SM protein Car/Vps33A regulates SNARE-mediated trafficking to lysosomes and lysosome-related organelles.. Mol Biol Cell.

[R2] Bellen HJ, Levis RW, Liao G, He Y, Carlson JW, Tsang G, Evans-Holm M, Hiesinger PR, Schulze KL, Rubin GM, Hoskins RA, Spradling AC (2004). The BDGP gene disruption project: single transposon insertions associated with 40% of Drosophila genes.. Genetics.

[R3] Brennwald P, Kearns B, Champion K, Keränen S, Bankaitis V, Novick P (1994). Sec9 is a SNAP-25-like component of a yeast SNARE complex that may be the effector of Sec4 function in exocytosis.. Cell.

[R4] Bridges Calvin B. (1937). Correspondences Between Linkage Maps and Salivary Chromosome Structure, as Illustrated in the Tip of Chromosome 2R of <i>Drosophila melanogaster</i>. CYTOLOGIA.

[R5] Brown JB, Boley N, Eisman R, May GE, Stoiber MH, Duff MO, Booth BW, Wen J, Park S, Suzuki AM, Wan KH, Yu C, Zhang D, Carlson JW, Cherbas L, Eads BD, Miller D, Mockaitis K, Roberts J, Davis CA, Frise E, Hammonds AS, Olson S, Shenker S, Sturgill D, Samsonova AA, Weiszmann R, Robinson G, Hernandez J, Andrews J, Bickel PJ, Carninci P, Cherbas P, Gingeras TR, Hoskins RA, Kaufman TC, Lai EC, Oliver B, Perrimon N, Graveley BR, Celniker SE (2014). Diversity and dynamics of the Drosophila transcriptome.. Nature.

[R6] Camacho C, Coulouris G, Avagyan V, Ma N, Papadopoulos J, Bealer K, Madden TL (2009). BLAST+: architecture and applications.. BMC Bioinformatics.

[R7] Chang YC, Gao Y, Lee JY, Peng YJ, Langen J, Chang KT (2024). Identification of secretory autophagy as a mechanism modulating activity-induced synaptic remodeling.. Proc Natl Acad Sci U S A.

[R8] Cook RK, Christensen SJ, Deal JA, Coburn RA, Deal ME, Gresens JM, Kaufman TC, Cook KR (2012). The generation of chromosomal deletions to provide extensive coverage and subdivision of the Drosophila melanogaster genome.. Genome Biol.

[R9] Dean DM, Maroja LS, Cottrill S, Bomkamp BE, Westervelt KA, Deitcher DL (2015). The wavy Mutation Maps to the Inositol 1,4,5-Trisphosphate 3-Kinase 2 (IP3K2) Gene of Drosophila and Interacts with IP3R to Affect Wing Development.. G3 (Bethesda).

[R10] Dean Derek M., Deitcher Derek L., Loehlin David W., Banta Lois M. (2020). Mapping a Mutation to its Gene: The "Fly Lab" as a Modern Research Experience. CourseSource.

[R11] Dean DM, Deitcher DL, Paster CO, Xu M, Loehlin DW (2022). "A fly appeared": sable, a classic Drosophila mutation, maps to Yippee, a gene affecting body color, wings, and bristles.. G3 (Bethesda).

[R12] Fasshauer D, Sutton RB, Brunger AT, Jahn R (1998). Conserved structural features of the synaptic fusion complex: SNARE proteins reclassified as Q- and R-SNAREs.. Proc Natl Acad Sci U S A.

[R13] Gloor, G., and W. Engels, 1992 Single fly preps for PCR. *Drosophila* Information Service 71: 148-149.

[R14] Gloor GB, Preston CR, Johnson-Schlitz DM, Nassif NA, Phillis RW, Benz WK, Robertson HM, Engels WR (1993). Type I repressors of P element mobility.. Genetics.

[R15] Grant P, Maga T, Loshakov A, Singhal R, Wali A, Nwankwo J, Baron K, Johnson D (2016). An Eye on Trafficking Genes: Identification of Four Eye Color Mutations in Drosophila.. G3 (Bethesda).

[R16] Haberman A, Williamson WR, Epstein D, Wang D, Rina S, Meinertzhagen IA, Hiesinger PR (2012). The synaptic vesicle SNARE neuronal Synaptobrevin promotes endolysosomal degradation and prevents neurodegeneration.. J Cell Biol.

[R17] Harbury PA (1998). Springs and zippers: coiled coils in SNARE-mediated membrane fusion.. Structure.

[R18] Itakura E, Kishi-Itakura C, Mizushima N (2012). The hairpin-type tail-anchored SNARE syntaxin 17 targets to autophagosomes for fusion with endosomes/lysosomes.. Cell.

[R19] Jenkins VK, Larkin A, Thurmond J, FlyBase Consortium (2022). Using FlyBase: A Database of Drosophila Genes and Genetics.. Methods Mol Biol.

[R20] Jiang P, Nishimura T, Sakamaki Y, Itakura E, Hatta T, Natsume T, Mizushima N (2014). The HOPS complex mediates autophagosome-lysosome fusion through interaction with syntaxin 17.. Mol Biol Cell.

[R21] Kahsai L, Cook KR (2018). Mapping Second Chromosome Mutations to Defined Genomic Regions in Drosophila melanogaster.. G3 (Bethesda).

[R22] Khvotchev M, Soloviev M (2022). SNARE Modulators and SNARE Mimetic Peptides.. Biomolecules.

[R23] Leader DP, Krause SA, Pandit A, Davies SA, Dow JAT (2018). FlyAtlas 2: a new version of the Drosophila melanogaster expression atlas with RNA-Seq, miRNA-Seq and sex-specific data.. Nucleic Acids Res.

[R24] Lee T, Luo L (1999). Mosaic analysis with a repressible cell marker for studies of gene function in neuronal morphogenesis.. Neuron.

[R25] Li M, Feng F, Feng H, Hu P, Xue Y, Xu T, Song E (2022). VAMP4 regulates insulin levels by targeting secretory granules to lysosomes.. J Cell Biol.

[R26] Lindsley, D. L., and G. G. Zimm, 1992 *The genome of Drosophila melanogaster* . Academic Press, San Diego.

[R27] Lloyd V, Ramaswami M, Krämer H (1998). Not just pretty eyes: Drosophila eye-colour mutations and lysosomal delivery.. Trends Cell Biol.

[R28] Lőrincz P, Takáts S, Kárpáti M, Juhász G (2015). iFly: The eye of the fruit fly as a model to study autophagy and related trafficking pathways.. Exp Eye Res.

[R29] Lucchesi JC (1968). Synthetic lethality and semi-lethality among functionally related mutants of Drosophila melanfgaster.. Genetics.

[R30] Morelli E, Ginefra P, Mastrodonato V, Beznoussenko GV, Rusten TE, Bilder D, Stenmark H, Mironov AA, Vaccari T (2014). Multiple functions of the SNARE protein Snap29 in autophagy, endocytic, and exocytic trafficking during epithelial formation in Drosophila.. Autophagy.

[R31] Morelli E, Mastrodonato V, Beznoussenko GV, Mironov AA, Tognon E, Vaccari T (2016). An essential step of kinetochore formation controlled by the SNARE protein Snap29.. EMBO J.

[R32] Munn K, Steward R (2000). The shut-down gene of Drosophila melanogaster encodes a novel FK506-binding protein essential for the formation of germline cysts during oogenesis.. Genetics.

[R33] Nonet ML, Staunton JE, Kilgard MP, Fergestad T, Hartwieg E, Horvitz HR, Jorgensen EM, Meyer BJ (1997). Caenorhabditis elegans rab-3 mutant synapses exhibit impaired function and are partially depleted of vesicles.. J Neurosci.

[R34] Ooi CE, Moreira JE, Dell'Angelica EC, Poy G, Wassarman DA, Bonifacino JS (1997). Altered expression of a novel adaptin leads to defective pigment granule biogenesis in the Drosophila eye color mutant garnet.. EMBO J.

[R35] Öztürk-Çolak A, Marygold SJ, Antonazzo G, Attrill H, Goutte-Gattat D, Jenkins VK, Matthews BB, Millburn G, Dos Santos G, Tabone CJ, FlyBase Consortium (2024). FlyBase: updates to the Drosophila genes and genomes database.. Genetics.

[R36] Rao SS, Stewart BA, Rivlin PK, Vilinsky I, Watson BO, Lang C, Boulianne G, Salpeter MM, Deitcher DL (2001). Two distinct effects on neurotransmission in a temperature-sensitive SNAP-25 mutant.. EMBO J.

[R37] Saifee O, Wei L, Nonet ML (1998). The Caenorhabditis elegans unc-64 locus encodes a syntaxin that interacts genetically with synaptobrevin.. Mol Biol Cell.

[R38] Sevrioukov EA, He JP, Moghrabi N, Sunio A, Krämer H (1999). A role for the deep orange and carnation eye color genes in lysosomal delivery in Drosophila.. Mol Cell.

[R39] Simpson F, Peden AA, Christopoulou L, Robinson MS (1997). Characterization of the adaptor-related protein complex, AP-3.. J Cell Biol.

[R40] Solinger JA, Spang A (2013). Tethering complexes in the endocytic pathway: CORVET and HOPS.. FEBS J.

[R41] Vilinsky I, Stewart BA, Drummond J, Robinson I, Deitcher DL (2002). A Drosophila SNAP-25 null mutant reveals context-dependent redundancy with SNAP-24 in neurotransmission.. Genetics.

[R42] Yang Y, Kim J, Kim HY, Ryoo N, Lee S, Kim Y, Rhim H, Shin YK (2015). Amyloid-β Oligomers May Impair SNARE-Mediated Exocytosis by Direct Binding to Syntaxin 1a.. Cell Rep.

[R43] Yang X, Tu W, Gao X, Zhang Q, Guan J, Zhang J (2023). Functional regulation of syntaxin-1: An underlying mechanism mediating exocytosis in neuroendocrine cells.. Front Endocrinol (Lausanne).

